# Electrochemical Detection of *p*-Aminophenol by Flexible Devices Based on Multi-Wall Carbon Nanotubes Dispersed in Electrochemically Modified Nafion

**DOI:** 10.3390/s140508926

**Published:** 2014-05-21

**Authors:** Graziella Scandurra, Arena Antonella, Carmine Ciofi, Gaetano Saitta, Maurizio Lanza

**Affiliations:** 1 Department of Electronic Engineering, Chemistry and Industrial Engineering, Messina University, Messina 98166, Italy; E-Mails: gscandurra@unime.it (G.S.); cciofi@unime.it (C.C.); saitta@unime.it (G.S.); 2 CNR, Institute for Chemical-Physics Processes, Messina Section, Messina 98166, Italy; E-Mail: lanza@its.me.cnr.it

**Keywords:** *p*-aminophenol, electrochemical sensor, Nafion/MWCNTs-modified electrodes

## Abstract

A conducting composite prepared by dispersing multi-walled carbon nanotubes (MWCNTs) into a host matrix consisting of Nafion, electrochemically doped with copper, has been prepared, characterized and used to modify one of the gold electrodes of simply designed electrochemical cells having copier grade transparency sheets as substrates. Electrical measurements performed in deionized water show that the Au/Nafion/Au-MWCNTs–Nafion:Cu cells can be successfully used in order to detect the presence of *p*-aminophenol (PAP) in water, without the need for any supporting electrolyte. The intensity of the redox peaks arising when PAP is added to deionized water is found to be linearly related to the analyte in the range from 0.2 to 1.6 μM, with a detection limit of 90 nM and a sensitivity of 7 μA·(μM^−1^)·cm^−2^.

## 1. Introduction

Aminophenols are well known aromatic compounds that exist in three isomeric forms differing from each other by the sites at which the oxidizable NH_2_ and OH groups are positioned around the benzene ring. The isomer having the amino group in the *para*- position, known as *p*-aminophenol (PAP), is widely used as precursor and intermediate in a variety of chemical syntheses. As a consequence of this, PAP and its derivatives are among the most common pollutants, toxic to aquatic life, found in effluent wastes from oil refineries, rubbers, dyes, lubricants, textiles, and pharmaceuticals production and processing [1]. Exposure to PAP and PAP ingestion must be limited, as there are strong evidences that *p*-aminophenol has toxic or allergenic effects on skin and that it has nephrotoxicity and teratogenic effects harmful for humans, animals, and plants. Being PAP the primary hydrolytic degradation product of paracetamol, an analgesic and antipyretic that is among the most marketed and consumed around the world, to prevent harmful effects of ingestion, its presence in the drug substances is limited to a level of 50 ppm (0.005% w/w) by the European, United States, British and German Pharmacopoeias [2]. Exposure to PAP can be the consequence of hair colouring usage as well, since *p*-aminophenol is contained in many cosmetic preparations [3]. In addition, it is found that the concentration of PAP in urine samples can be a useful biomarker to check the health of workers exposed to aniline [4]. The need for techniques capable to detect *p*-aminophenol in a reliable way is therefore well recognized. Such a task can be fulfilled by using high performance chromatography, when dealing with concentrations well below the micromolar range [5]. Capillary electrophoresis [6] and optical chemical sensors [7] can be used as well. The detection of PAP in a wide concentration range and with detection limits usually above 10^−8^ M can be achieved by routine electrochemical sensing [8–13], taking advantage of the electrochemically active nature of PAP, known to undergo electron/proton processes in aqueous environment, yielding *p*-benzoquinone as the final oxidation product [14]. Compared to the other techniques, electrochemical sensing allows fast and selective detection of PAP, avoiding time-consuming procedures and extraction processes, and does not require any complex and expensive apparatus. In this paper, we demonstrate that the presence of PAP in water can be electrochemically detected with a detection limit below 100 nM, without using any supporting electrolyte. Aimed at this, and following the same approach employed in a previous paper [15], we developed a solid state electrochemical sensor on a plastic substrate. The sensor is based on an ion conducting membrane applied on the top of thermally evaporated gold electrodes, with one of the electrodes modified using an ink prepared by electrochemically dissolving metallic copper into Nafion, and then by dispersing MWCNTs into the Nafion:Cu host. It is found that, in response to PAP, broad peaks arise in the voltage-current cycles of the gold/Nafion/MWNT-Nafion:Cu devices, measured in water. The current peak intensity is linearly related to the PAP content, in the concentration interval ranging between 2 × 10^−7^ M and 16 × 10^−7^ M.

## 2. Experimental Section

### 2.1. Preparation of the MWNTs-Nafion:Cu Composite

**Figure 1.**
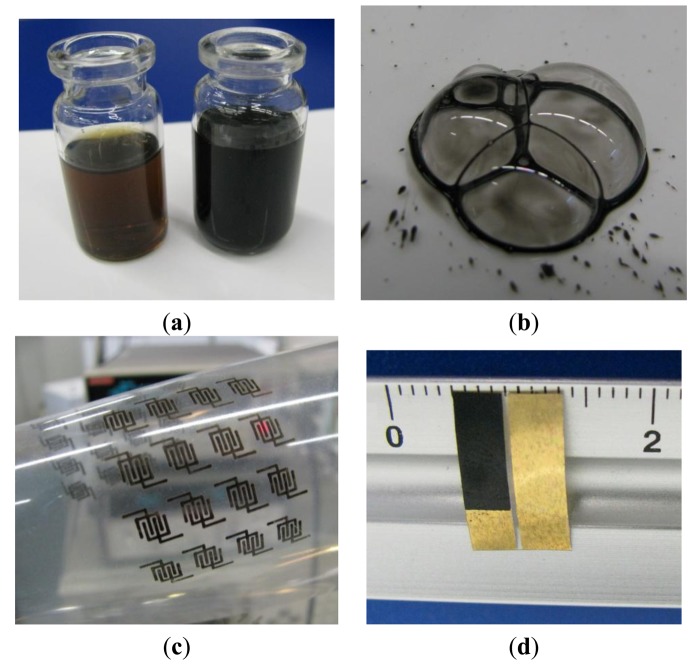
(**a**) Dark brown and black dispersions of Nafion:Cu and of Nafion:Cu-MWCNTs mix0ture; (**b**) drops of Nafion:Cu-MWCNTs show how carbon nanotubes are dispersed in the mix0ture; (**c**) interdigitated conducting patterns printed from the Nafion:Cu-MWCNTs ink; (**d**) a typical example of the developed Au/Nafion/MWNTs-Nafion:Cu/Au sensors.

Nafion 117 dispersion in a mixture of water and alcohol from IonPower (New Castle, DE, US), copper wire from Aldrich (St. Louis, MO, US) and MWCNTs having 5–20 nm outer diameter and length ranging between 1 μm and 10 μm, purchased from PlasmaChem (Berlin, Germany), were used as received. Nafion films deposited from the above mixture on top of copper metalized plastic substrates are found to slowly dissolve the underlying metal. Starting from this observation, aimed at doping Nafion, copper electrodes were placed inside a beaker containing Nafion 117 alcoholic dispersion mixed with deionized water, and an electric field of about 10 V/cm was applied between the electrodes. After a few hours, it became apparent that, while the copper cathode was consuming, the uncoloured transparent mixtures turned to pale green first, and then started to darken, until assuming a brown colour (see Figure 1a).

The same kind of result could be simply achieved after a few weeks with the thin copper wires at rest into initially transparent and uncoloured Nafion dispersions. MWCNTs were then dispersed into the electrochemically prepared Nafion:Cu mixtures, at a ratio of 50 mg/10 mL. After adding suitable amounts of ethanol/water, prolonged sonication yielded a dark and clear MWCNTs/Nafion:Cu ink (Figure 1a,b), from which conducting films and patterns could be easily applied by means of spin coating or inkjet printing (see Figure 1c).

### 2.2. Preparation of the Au/Nafion/MWNT-Nafion:Cu/Au Electrochemical Device

Simple electrochemical devices were obtained starting from rectangular parallel gold electrodes, spaced by half a millimeter, thermally evaporated under vacuum onto copier grade transparency sheets (Tartan, St. Paul, MN, US). The MWCNTs-Nafion:Cu ink was then applied on the top of one of the gold electrodes. After all the residual solvent evaporated, the device was completed by depositing a thin Nafion layer onto both the gold and on the Au/MWCNTs-Nafion:Cu electrode (a typical example of the resulting devices is shown in Figure 1d). Several batches of carbon nanotube and Nafion:Cu electrodes were prepared starting form copper saturated Nafion dispersions. Although the morphology of each batch has not been systematically investigated by means of SEM analysis, all samples (in the order of several tens of units) belonging to different batches and prepared at different times have always resulted in the same experimental results as far as the electrochemical behavior and the sensitivity toward PAP are concerned.

### 2.3. Materials and Device Characterization

The morphology of the Nafion:Cu membrane and of conducting films developed from the MWCNTs-Nafion:Cu ink, is investigated by Scanning Electron Microscopy (SEM) measurements performed using a 5600LV electron microscope (JEOL, Peabody, MA, US).

The electrical properties of the Nafion:Cu membrane are characterized by means of impedance measurements, performed in air by means of an Agilent 4284A LCR meter (Agilent Technologies, Santa Clara CA, US), in the frequency range between 20 Hz and 1 MHz, using a 100 mV amplitude sinusoidal input. Time domain electrical characterization of the Au/Nafion/MWNT-Nafion:Cu/Au cells is performed using the 2400 source meter (Keithley, Cleveland, OH, US) by measuring the current flowing through the device, in response to triangular voltage inputs. Measurements are carried out at different voltage time rates, over symmetric voltage windows. Sensing tests are performed with the sensors immersed in deionized water. Diluted PAP solutions are injected by using a micro syringe into the solution containing the sensor under test, in order to increase the analyte concentration in steps of 2 × 10^−7^ M. To avoid the occurrence of degradation processes, due to the limited photostability of *p*-aminophenol (evidenced by a detectable change of the colour of the solution after a few days of exposure to daylight), all the sensing tests are performed in the dark, and with freshly prepared solutions.

## 3. Results and Discussion

**Figure 2.**
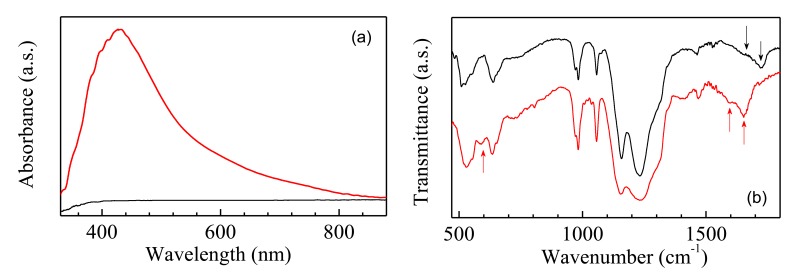
(**a**) Optical absorbance spectrum of a Nafion film (black) and of a Nafion:Cu film (red). (**b**) Infrared transmittance spectrum of a Nafion film (black), and of a Nafion:Cu film (red) deposited on silicon substrates.

The electrochemical dissolution of copper electrodes immersed into aqueous solution of Nafion under an externally applied electric field, as it is described in the previous section, involves the formation of hydroxides species at the metallic cathode. Such copper hydroxides may be converted into the red cuprous oxide (Cu_2_O) and into the black cupric oxide (CuO), the presence of which, with a predominance of the former one, may be responsible for the brownish color of the resulting Nafion:Cu mixtures. In addition, the copper ions may exchange the Nafion H^+^ protons into the Nafion matrix. As it is shown in Figure 2a, the broad and intense absorption band that appears at approximately at 420 nm in the spectrum of a thin Nafion:Cu film is responsible for the color change upon doping, while the spectrum of the optically transparent Nafion film is completely flat in the visible range. On the other hand, the infrared transmittance spectra of Nafion and of Nafion:Cu thin films are very similar to one another, as it is shown in Figure 2b, thus indicating that Nafion is not degraded or altered by the electric field applied to prepare the Nafion:Cu mixture.

Comparing the two spectra of Figure 2b, the small structure at about 595 cm^−1^ in the Nafion:Cu spectrum (red curve), not found in the Nafion spectrum (black curve), is positioned in the spectral region where the stretching of Cu_2_O or CuO species is usually observed [16]. This finding could be the evidence that copper oxides are loaded into the Nafion host matrix. In addition, comparison between the spectra reported in Figure 2b shows that the broad band and the shoulder positioned at about 1720 cm^−1^ and 1650 cm^−1^ in the Nafion spectrum (marked with arrows), are shifted towards lower wavenumbers region in the spectrum of Nafion:Cu. Responsible of such a spectral change can be the bending of bulk water [17], the band of which, usually peaked at approximately 1645 cm^−1^, shifts toward higher wavenumbers as the water content increases, while it shifts towards lower wavenumbers (as it is observed in our case) in situations where the hydrogen bonding network is weakened by the interactions between the water molecules and the metal cations inserted into the Nafion matrix.

**Figure 3.**
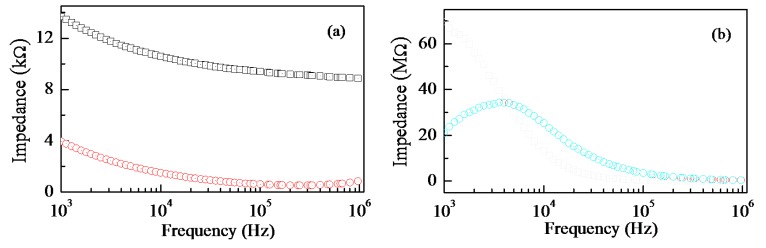
(**a**) Frequency dependence of the real part (black squares) and of the opposite of the imaginary part (red circles) of the complex impedance of a Nafion film. (**b**) Frequency dependence of the real part (black squares) and of the opposite of the imaginary part (red circles) of the complex impedance of a Nafion:Cu film.

From the electrical point of view, the electrochemical doping of copper into Nafion has remarkable effects, as the results of frequency resolved impedance measurements plotted in Figure 3 clearly show. The data of Figure 3 refer to thin Nafion and thin Nafion:Cu films, deposited onto the gap between gold electrodes spaced by about half a mm. It can be noticed that both the real and the opposite of the imaginary part of the impedance of Nafion:Cu are orders of magnitude larger than those of Nafion. Such an increase, and the appearance of a relaxation process denoted by the peak in the opposite of the imaginary part of the complex impedance of Nafion:Cu (Figure 3b) plotted *versus* frequency, can be both regarded as consequences of the replacement of the highly conducting protons H^+^ with the copper cations.

**Figure 4.**
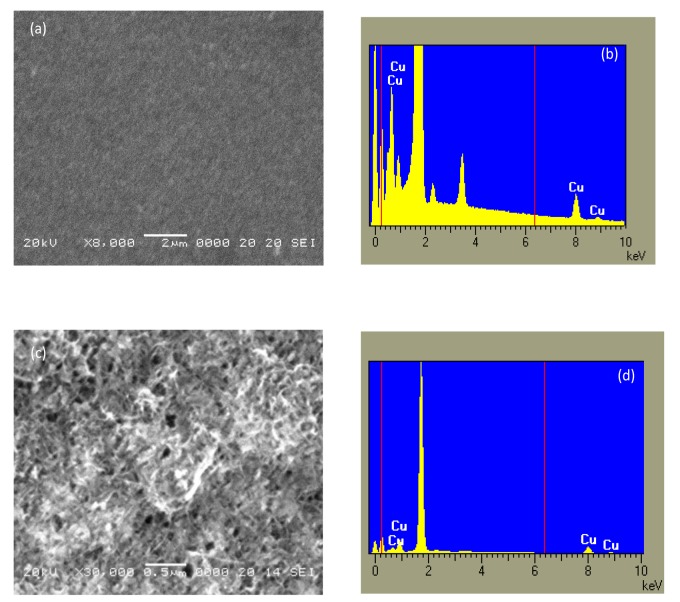
SEM micrography and elemental composition as deduced by EDX of a Nafion:Cu film (**a**,**b**), and of a film deposited from the Nafion:Cu-MWCNTs mixture (**c**,**d**).

Figure 4a,b shows the SEM image and the elemental composition as deduced from the EDX analysis of a typical Nafion:Cu film deposited onto a silicon substrate. The morphology of the film is not as smooth as a polymer surface is expected to be, revealing the presence of granules that may be copper oxide nanoparticles aggregations. Films deposited from the dispersions of MWCNTs into the Nafion:Cu host, as the one shown in the micrograph Figure 4c, have a complex morphology, predominated by disordered carbon nanotubes networks into which small particles seem to be entrapped. According to the EDX analysis (Figure 4d), these particles contain copper.

**Figure 5.**
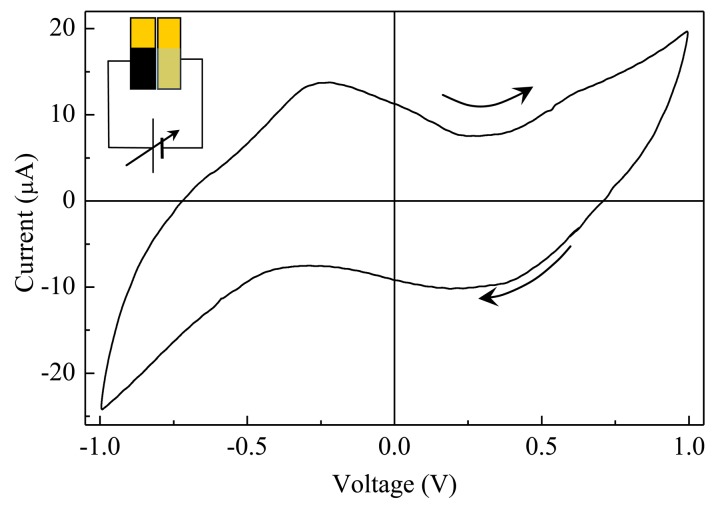
Steady state current-voltage loop of a typical Au/Nafion/MWNTs-Nafion:Cu/Au cell in deionized water. The scan rate is 50 mV/s. The inset shows the polarity of the voltage.

The electrical behaviour of Au/Nafion/MWNT-Nafion:Cu/Au cells immersed in deionized water has been tested using zero average triangular voltage inputs with 40 s period. After a transient phase in which the current initially grows with the increasing voltage, the current starts to cycle, forming clockwise loops having a shape that slightly changes cycle after cycle, until a steady state is reached, in which the current plotted against the voltage forms a stable loop, as the one shown in Figure 5. Current-voltage loops arise whenever the current is not only affected by the applied voltage, but it also depends on the time rate at which the voltage changes. Such kind of a situation is common when dealing with capacitive-resistive systems, and in the presence of electroactive species undergoing redox processes over the swept voltage window. In the case of the Au/Nafion/MWNTs-Nafion:Cu/Au cell, capacitive effects may arise from the double layer capacitance that forms at the interface between the electrodes and the ion conducting polymer. At the same time, redox transitions may also occur, involving the copper ions entrapped into the Nafion network. The current-voltage loop of Figure 5 is slightly asymmetrical, with a distinct forward current peak positioned at about −230 mV and a less resolved reverse current peak at 240 mV, approximately. Separation between the forward and the reverse current peaks and the ratio between the reverse and the forward current peak intensity lower than unity, suggest that the current peaks originate from irreversible redox transition. Such peaks, that are not observed with electrodes obtained by dispersing carbon nanotubes in Nafion without dissolving copper in the host matrix, are possibly due to processes involving Cu, CuO, Cu(OH), CuO_2_ and Cu(OH)_2_ species with the metal changing its oxidation state from 0 to 1 and to 2 [18].

In order to evidence the role of Nafion on the electrode, we performed voltage current cycles using samples whose electrodes were obtained with a mixture of carbon nanotubes and copper oxide nanoparticles dispersed into a hydroxypropylcellulose matrix. In this situation it has been observed that the voltage current cycles, for the same operating conditions, enclose a smaller area with respect to that reported in Figure 5, with lower current levels. Samples with Nafion free electrodes did non evidenced any response to the PAP within the explored concentration ranges. Therefore, we conclude that the Nafion matrix has a major role in obtaining the PAP sensitive electrode, this role possibly being related to the predominant oxidation state of the metal that in the Nafion:Cu composite appears to be present as Cu(I).

**Figure 6.**
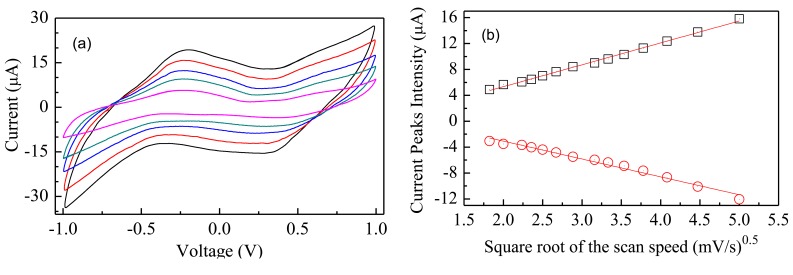
(**a**) Steady state current-voltage cycles of a typical Au/Nafion/MWNTs-Nafion:Cu/Au cell, measured over the same voltage range in pure deionized water, at different scan rates. (**b**) Forward (squares) and reverse current peaks intensity (circles), plotted as a function of the square root of the scan rate. The solid lines represent the results of linear fitting analysis.

Figure 6a shows the results of current-voltage measurements performed at different scan rates. It can be noticed that forward and reverse current peaks separation increases, while their intensity grows, as the scan rate increases. The plots of Figure 6b show that the intensity of the forward and reverse current peaks is linearly related to the square root of the scan rate, as it always happens when the redox process is controlled by diffusion. In our case, diffusion phenomena are possibly the result of the concentration gradient of the electrochemically active copper inserted into the Nafion host matrix.

**Figure 7.**
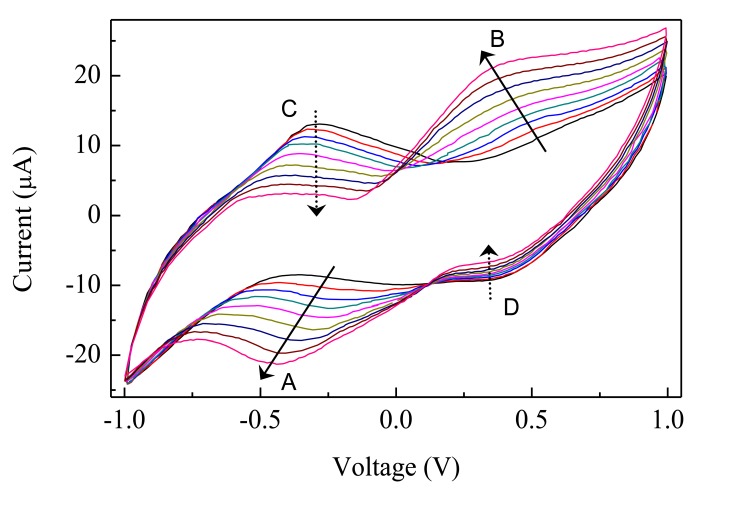
Current-voltage cycles of a typical Au/Nafion/MWNTs-Nafion:Cu/Au cell Gold/Nafion:Polypyrrole/MWCNTs device, measured in distilled deionized water (black line), and in the presence of PAP that increases in steps of 2 × 10^−7^ M. Scan rate is 50 mV/s. The dashed arrows indicate how the original current peaks progressively decrease with exposure to increasing analyte concentration. The solid arrows indicate the new peaks that arise and grow in response to increased PAP concentration.

**Figure 8.**
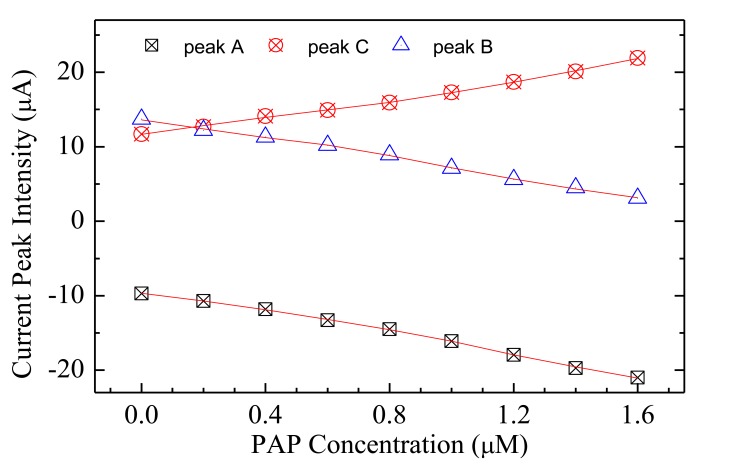
Intensity of the current peaks (identified as A, B and C in the plot of Figure 7, from which data are extracted) of a typical Au/Nafion/MWNTs-Nafion:Cu/Au, as a function of the PAP concentration.

The steady state current-voltage loops of Figure 7 show how the electrical response of a typical Au/Nafion/MWNT-Nafion:Cu/Au cell immersed in deionized water changes in response to *p*-aminophenol added in subsequent steps of 200 nM. The exposure to increasing amounts of PAP has the effect of decreasing the original forward and reverse current peaks (marked C and D and indicated by the dashed arrows in Figure 7), in favour of a new couple of broad peaks (marked A and B and indicated by the solid arrows in Figure 7). The intensity of these latter peaks is found to increase progressively, and their position is observed to shift towards the lower voltage side, as the PAP concentration increases. If the water containing PAP is replaced by pure deionized water, the peaks marked with the solid arrows progressively reduce, and after a transient phase, the current-voltage loop goes back to its original shape, with forward and reverse current peaks positioned at about −230 mV and at 240 mV, respectively. The way the intensity of the peaks A, B, and C in Figure 7 changes in response to PAP is shown in Figure 8. It can be noticed that peaks A and B linearly grow with PAP concentration, while peak C decreases: further, it seems that the rate at which the peak marked as B grows is the same rate at which peak C decreases. The sensitivity toward PAP that can be estimated from the slope of the linear calibration curves of Figure 8b is of the order of 7 μA·(μM^−1^)·cm^−2^, while the limit of detection is equal to 90 nM.

The detection limit of the developed sensor is compared to that of other sensors reported in the literature in Table 1. It must be noted that all the other sensors require a supporting electrolyte (listed in the table) whose nature and concentration has been tailored in order to maximize the sensor performances. As stressed before, in our case there is no need for any supporting electrolyte, that has to be regarded as a significant advantage, because of the very design of the sensor.

**Figure 9.**
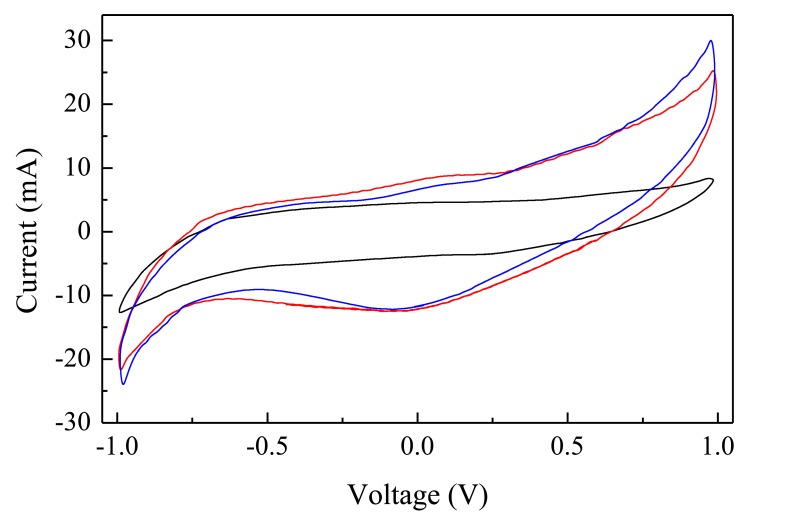
Current-voltage cycles measured in the same operating conditions used for the other sensing tests, in deionized water and in the presence of 5 μM hydrogen peroxide (black line), of 5 μM ammonia (red) and of 5 μM ascorbic acid (blue).

In order to investigate the sensor selectivity, sensing tests are performed in the presence of paracetamol, glucose, hydrogen peroxide, ammonia, and ascorbic acid. The presence of paracetamol does not result in any observable effect in concentrations up to 10 μM, and the same is true for glucose. In the presence of hydrogen peroxide, at concentrations in the μM range, the area subtended by the current-voltage cycle decreases, with lower current, and the peaks of Figure 5 disappear. The effect of the presence of analytes such as ascorbic acid and ammonia, at concentrations in the μM range, is a higher current (that is the result of the conductivity increase of the solution), the disappearance of the peaks of Figure 5, and the appearance of new peaks that do not superimpose to those due to the presence of PAP. These results, illustrated in Figure 9, indicate that the sensor has good selectivity towards PAP.

**Figure 10.**
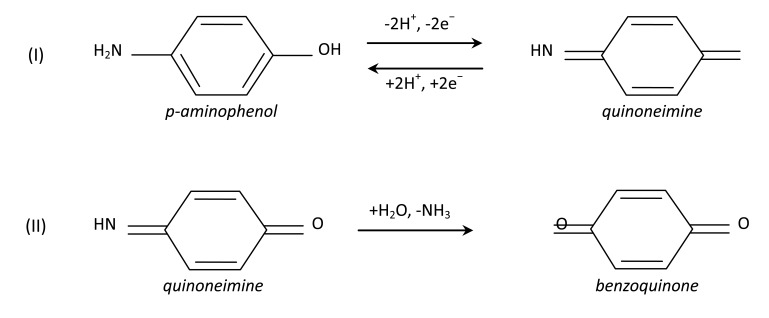
(**I**) Redox transition from PAP to quinoneimine. (**II**) conversion from quinoneimine (the intermediate product of PAP degradation) to benzoquinone, one of the possible final products of degradation.

Electrochemical detection of PAP in water exploits the electrochemical activity of p-aminophenol, known to undergo oxidation, forming quinoneimine, which can in turn be reduced back to PAP, following Scheme I of Figure 10. Such a kind of redox transition, in a variety of pH conditions, in the presence of suitable supporting electrolytes, and on suitably modified bare electrodes, produces a couple of quasi-reversible redox peaks, found to be spaced by about a few hundreds of mV at scan rates on the order of 50 mV/s. These latter findings seem to be rather different, compared to the redox features of Figure 8b. In order to understand such a difference, it may be of interest to investigate on the catalytic role that Nafion:Cu seems to exert on PAP degradation. PAP in itself is a rather unstable species, that easily undergo decomposition either in the powder form, when exposed to air and moisture, and in aqueous solution [14]. The rate at which degradation takes place, and the nature of degradation products between the original PAP molecules and the final degradation product depend on a variety of factors including the temperature, the exposure to light, the pH condition, and the presence of possible catalysts. A possible oxidation scheme, illustrated in Figure 10 (Scheme II), leads to benzoquinone as a final product, through the formation of quinoneimine as intermediate.

**Figure 11.**
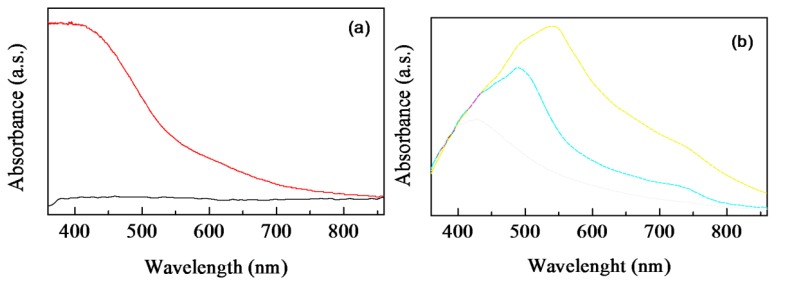
(**a**) Absorbance of a water solution of PAP as prepared (black), and after few weeks ageing in air (red); (**b**) absorbance of Nafion:Cu in a water ethanol mixture before (black), and half one hour after the addition of 10 mM PAP (red). The blue line is the steady state absorbance of the same Nafion:Cu solution with 10 mM PAP, after two hours of staying in air.

Formation of benzoquinone could be responsible of the degradation of PAP left in neutral deionized water, stored in air for weeks, that is evidenced by the slow transition from the transparent uncoloured as prepared solutions to the dark yellow colour of the solutions aged for weeks, revealed by spectrophotometric measurements of Figure 11a. Different products and accelerated degradation rates are observed in the presence of suitable catalyzers. Semiconducting copper oxide [19], and titanium dioxide [20] incorporated into zeolite, for instance, have been found to act as photocatalyzers, increasing the degradation of PAP in water solution. We find that the Nafion-Cu composite developed by us also has similar effects. As an evidence of this, Figure 11b compares the absorbance of Nafion:Cu dispersed in a mixture of water and ethanol, measured before, half one hour and two hours after a few drops of water solution of PAP 10 mM have been added to the Nafion:Cu dispersion. Immediately after the addition of PAP, it became apparent that new absorption arose above 450 nm, in the visible spectral region, where either freshly deposited and aged PAP solutions are transparent, and superimposes over the original absorption band ascribable to Nafion:Cu. This finding suggests that in the presence of Nafion-Cu, degradation starts immediately, as PAP is kept in contact with Nafion:Cu. While the degradation proceeds, the Cu:Nafion-PAP mixture turns its color from the initial dark brown to the dark red. The absorbance of the stable product that results from the degradation of PAP in the presence of Nafion:Cu (see the blue line of Figure 11b) is characterized by the presence of a very strong absorbance peak, centred at about 550 nm, similar to that observed in the case of the purple dye found as degradation product of PAP at low pH, in the presence of peroxidic agents [14].

Likely, not only the Nafion:Cu mixture, but the Nafion:Cu-MWCNTs composite also has the ability to catalyze the PAP degradation. If this is the case, the couple of redox peaks, the intensity of which seems to be linearly related to PAP concentration, would not be related to the redox transition schematically illustrated in Scheme I of Figure 10, but they could be ascribable to the violet species that forms as the result of the PAP degradation catalyzed by Nafion:Cu. The catalytic activity of Nafion:Cu could therefore explain the remarkable sensitivity towards PAP, and at the same time could account for the difference between the redox peaks arising in response to PAP observed here and those reported in the case of other PAP electrochemical sensors [10–13].

## 4. Conclusions

It is demonstrated that resistive-capacitive electrochemical devices, using Nafion as solid electrolyte and having electrodes consisting of a mixture of carbon nanotubes and Nafion doped with copper, can be successfully used to detect the presence of *p-*aminophenol in water, at concentrations below the micromolar range, without the need of any supporting electrolyte. The detection mechanism is found to be related to the catalytic activity of the Nafion:Cu matrix on the PAP photodegradation, evidenced by the spectrophotometric investigation.

## Figures and Tables

**Table 1.** Detection limit of our sensor compared to that of other electrochemical sensors.

	**Supporting Electrolyte**	**Detection Limit**	**Ref.**
This work	none	90 nM	–
hemin-modified molecularly imprinted polymer	0.05 mol·L^−1^ TRIS buffer solution	3.0 μM	[8]
graphene–polyaniline modified glassy carbon electrode	0.1 M phosphate buffer solutions	65 nM	[10]
CTAB Modified Carbon Paste Electrode	0.1 M phosphate buffer solutions	100 nM	[12]
(PEDOT)-modified glassy carbon electrode	phosphate buffer	1.2 μM	[13]
